# Occurrence and Risk Factors for New Dependency on Chronic Care, Respiratory Support, Dialysis and Mortality in the First Year After Sepsis

**DOI:** 10.3389/fmed.2022.878337

**Published:** 2022-05-19

**Authors:** Melissa Spoden, Christiane S. Hartog, Peter Schlattmann, Antje Freytag, Marlies Ostermann, Lisa Wedekind, Josephine Storch, Konrad Reinhart, Christian Günster, Carolin Fleischmann-Struzek

**Affiliations:** ^1^Research Institute of the Local Health Care Funds (AOK), Berlin, Germany; ^2^Department of Anesthesiology and Operative Intensive Care Medicine, Charité Universitätsmedizin Berlin, Berlin, Germany; ^3^Klinik Bavaria, Kreischa, Germany; ^4^Institute of Medical Statistics, Computer and Data Sciences, Jena University Hospital, Jena, Germany; ^5^Institute of General Practice and Family Medicine, Jena University Hospital, Jena, Germany; ^6^Department of Critical Care, King's College London, Guy's and St Thomas' Hospital, London, United Kingdom; ^7^Center for Sepsis Control and Care, Jena University Hospital/Friedrich Schiller University Jena, Jena, Germany; ^8^Institute of Infectious Diseases and Infection Control, Jena University Hospital, Jena, Germany

**Keywords:** sepsis, long-term mortality, risk factors, occurrence, post-sepsis syndrome

## Abstract

Sepsis survival is associated with adverse outcomes. Knowledge about risk factors for adverse outcomes is lacking. We performed a population-based cohort study of 116,507 survivors of hospital-treated sepsis identified in health claims data of a German health insurance provider. We determined the development and risk factors for long-term adverse events: new dependency on chronic care, chronic dialysis, long-term respiratory support, and 12-month mortality. At-risk patients were defined by absence of these conditions prior to sepsis. Risk factors were identified using simple and multivariable logistic regression analyses. In the first year post-sepsis, 48.9% (56,957) of survivors had one or more adverse outcome, including new dependency on chronic care (31.9%), dialysis (2.8%) or respiratory support (1.6%), and death (30.7%). While pre-existing comorbidities adversely affected all studied outcomes (>4 comorbidities: OR 3.2 for chronic care, OR 4.9 for dialysis, OR 2.7 for respiratory support, OR 4.7 for 12-month mortality), increased age increased the odds for chronic care dependency and 12-month mortality, but not for dialysis or respiratory support. Hospital-acquired and multi-resistant infections were associated with increased risk of chronic care dependency, dialysis, and 12-month mortality. Multi-resistant infections also increased the odds of respiratory support. Urinary or respiratory infections or organ dysfunction increased the odds of new dialysis or respiratory support, respectively. Central nervous system infection and organ dysfunction had the highest OR for chronic care dependency among all infections and organ dysfunctions. Our results imply that patient- and infection-related factors have a differential impact on adverse life changing outcomes after sepsis. There is an urgent need for targeted interventions to reduce the risk.

## Introduction

Sepsis, the dysregulated host response to infection (1), globally affects more than 49 million patients every year (2). In recent years, the number of sepsis survivors has risen following increased sepsis identification rates and improved survival in many industrialized countries (3, 4). Sepsis survival, however, is associated with new morbidity and increased mortality; hence sepsis has been described as a “hidden public health disaster” (5). Post-sepsis morbidity includes a broad range of new-onset physical, mental or cognitive impairments (6, 7). According to a nationwide US cohort of sepsis survivors aged 65 and older, one sixth experienced persistent physical disability and cognitive impairment, and one third died during the following year (8, 9). The World Health Organization has urged its member states to implement interventions to reduce long-term complications (10). However, there is a lack of epidemiological data and knowledge regarding the identification of high-risk patients and specific interventions (11). Previous studies mostly focused on risk factors for re-hospitalization and mortality (12–14), but data on factors associated with other patient-relevant adverse long-term outcomes are scarce (15).

Among the myriad of new diseases which can be associated with sepsis survival (6), new chronic care dependency, need for chronic dialysis and inability to breathe without mechanical support can be regarded as particularly crippling conditions. We explored the development of these adverse outcomes, the risk of mortality and the impact of corresponding risk factors using a large database of sepsis survivors identified in nationwide German health claims records of the AOK (Allgemeine Ortskrankenkasse) health insurance, which covers 30% of the German population (16). The study was registered (DRKS00016340) and approved by the local institutional review board of the Jena University Hospital, Germany (2019-1282-Daten).

## Materials and Methods

### Sepsis Survivors and Pre-defined Subgroups

The data source and sepsis survivor cohort have been described previously (16). In short, all patients older than 15 years with at least one inpatient stay with sepsis between 01/2013 and 12/2014 (index hospitalization) were identified among 23.0 million beneficiaries of the AOK health insurance. The diagnosis of sepsis was based on explicit International Statistical Classification of Diseases and Related Health Problems Version 10 German Modification (ICD-10-GM) codes (17) and included non-severe and severe sepsis (17) (Supplement in Supplementary Material). Patients with an in- or outpatient diagnosis of sepsis 24 months prior to the index hospitalization and patients without continuing insurance between 2009 and a three-year period following the index hospitalization or until death were excluded. Patients who survived their index hospitalization were included in the cohort of sepsis survivors and followed up for 12 months. Pre-defined subgroups were (1) survivors of severe sepsis [defined by ICD-10-GM code R65.1 (severe sepsis) and R57.2 (septic shock)] and non-severe sepsis, (2) survivors of intensive care unit (ICU)-treated sepsis [defined by procedural codes for intensive care complex treatment (Supplement in Supplementary Material)] and non-ICU-treated sepsis, and (3) survivors without any physical, cognitive or medical impairment in the 12 months prior to hospitalization with sepsis (Supplement in Supplementary Material).

### Identification of Adverse Outcomes and At-Risk Patients

The following adverse outcomes were analyzed: (1) new onset chronic care dependency defined as new need for nursing home residency or new nursing care level ≥2 in accordance with the German care level system. Level 2: “Substantial impairment of independence”, level 3: “Severe impairment” level 4: “Most severe impairment”, level 5: “Most severe impairment and need for special care”, these levels entitle patients to long-term care insurance benefits, (2) new dialysis dependence, and (3) new need for long-term respiratory support, and (4) 12-month mortality. (2) and (3) were defined as at least one in-patient and out-patient ICD- and procedural code (Supplement in Supplementary Material) in the 12 months period after hospital discharge.

**Figure 1:**
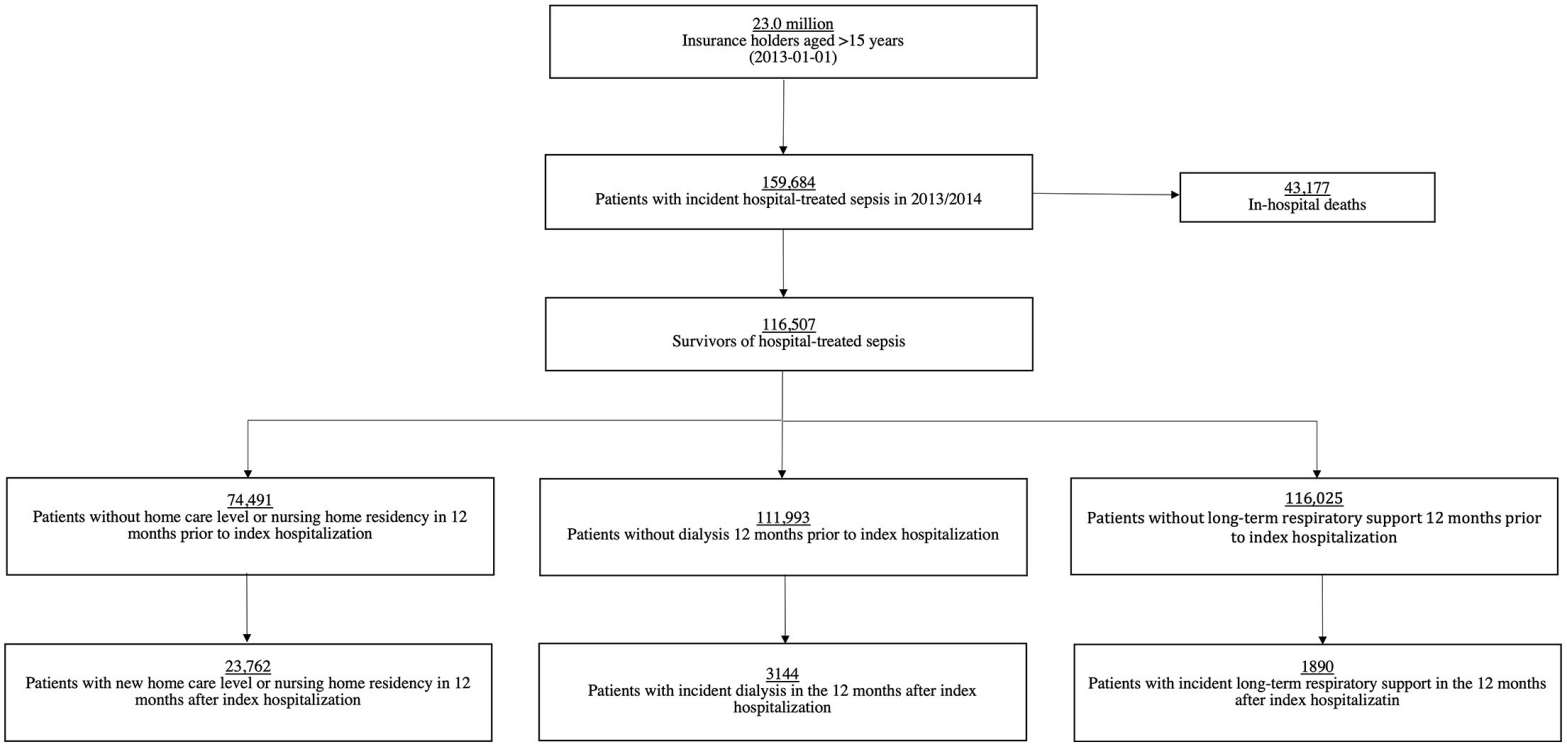
Flow of study inclusion.

Survivors at-risk were defined as not having the respective condition in the 12-month period prior to the index hospitalization (Figure 1). For example, survivors with any preexisting chronic care dependency within the 12 months period before the index hospitalization were excluded from the analysis exploring risk of chronic care dependency after sepsis. Our analyses include at-risk patients who died within the 12-month follow-up period.

### Definition of Potential Risk Factors

The selection of potential risk factors was based on a comprehensive literature search (12, 13, 18, 19). Thus, we included age, gender, number of pre-existing comorbidities according to the unweighted Charlson Comorbidity Index (CCI) (20), pre-existing immobility (e.g., dependency on wheelchair, need for assistance due to reduced mobility) within 12 months prior to sepsis, and pre-existing asplenia within 5 years prior to sepsis as potential patient-related risk factors. In addition, we included emergency admission, source of infection, hospital acquired infection, multi-resistant pathogen infection, organ dysfunction, and surgical treatment during index hospitalization as potential infection-related risk factors. A detailed description of ICD-10-GM and procedural codes used to define these covariates can be found in Supplement in Supplementary Material.

### Statistical Analyses

First, we investigated the occurrence of adverse events among all index hospitalization survivors and among the five pre-defined subgroups. We describe proportions and 95% Wilson confidence intervals. Chi-square tests were used for comparisons between subgroups.

Risk factors were analyzed for survivors at risk in the total survivor cohort. In a first step, the statistical significance between each potential risk factors and each of the four adverse outcomes were tested using univariate logistic regressions. All potential risk factors with *p*-values <0.20 in the univariate logistic regression analysis were included in the multivariable logistic regression models (21). To find the best predictive subset of potential risk factors while avoiding overfitting, we used a stepwise variable selection method with the Aikaike information criterion (AIC) (22). The patients' age and sex were specified as fixed predictors. Hence, these two variables were always included as predictors in all multivariable logistic regression models. For each outcome, the model including age, sex and the subset of potential risk factors with the lowest AIC was chosen as best fitting model. The stepwise AIC procedure was conducted in STATA using the GVSELECT package (23). Odds ratios (OR), *p*-value and 95% confidence intervals (CI) of both, simple and the best fitting multivariable logistic regression models are reported by outcome. All descriptive and risk factor analyses were performed using STATA version 16.0.

## Results

### Occurrence of Adverse Outcomes

Among 23.0 million beneficiaries, 159,684 sepsis patients were identified between 01/2013 and 12/2014, of whom 116,507 (73.0%) survived the index hospitalization (Figure 1). Demographics and clinical features of survivors are shown in Supplementary Table 1.

In total, 42.0% (*n* = 48,883) and 48.9% (*n* = 56,957) of all survivors had at least one new adverse outcome in the first six and twelve months post-sepsis, respectively (Table 1, Supplementary Table 2). At 12 months post-sepsis, 31.9% (*n* = 23,762 of 74,491 at-risk survivors) were newly dependent on chronic care, 2.8% (*n* = 3,144 of 111,993 at-risk survivors) were dialysis dependent, and 1.6% (*n* = 1,890 of 115,025 at-risk survivors) required long-term respiratory support. Twelve-month mortality was 30.7% (*n* = 35,765) among all survivors (Table 1).

**Table 1:** Occurrence of adverse outcomes 0–12 months after sepsis.

**Adverse outcome**	**All survivors**	**Patients w/o prior impairments**	**Severe sepsis**	**Non-severe sepsis**	***p*-value**	**ICU-treated sepsis**	**Non-ICU-treated sepsis**	***p*-value**
**No. of survivors**	116,507	8,622	37,840	78,667		32,238	84,269	
Survivors with ≥1 new adverse outcome among all survivors, % (95% CI)	48.9 (48.6–49.2)	29.8 (28.8–30.7)	54.8 (54.3–55.3)	46.0 (45.7–46.4)	<0.001	55.9 (55.3–56.4)	46.2 (45.9–46.6)	<0.001
New onset chronic care dependency among survivors w/o prior chronic care dependency, % (95% CI)	31.9 (31.6–32.2)	19.4 (18.6–20.2)	35.3 (34.7–35.9)	30.2 (29.8–30.6)	<0.001	37.5 (37.1–37.9)	29.4 (29.0–29.8)	<0.001
New onset dialysis dependency among survivors w/o prior dialysis, % (95% CI)	2.8 (2.7–2.9)	1.7 (1.5–2)	4.1 (3.9–4.3)	2.0 (1.9–2.1)	<0.001	4.7 (4.5–4.9)	2.1 (2.0–2.2)	<0.001
New onset respiratory support among survivors w/o prior respiratory support, % (95% CI)	1.6 (1.6–1.7)	1.0 (0.8–1.2)	2.5 (2.3–2.6)	1.2 (1.2–1.3)	<0.001	3.3 (3.1–3.5)	1.0 (0.9–1.1)	<0.001
12-months mortality among hospital survivors, % (95% CI)	30.7 (30.4–31)	15.2 (14.4–15.9)	33.9 (33.4–34.4)	29.2 (28.9–29.5)	<0.001	31.8 (31.3–32.3)	30.3 (30–30.6)	<0.001

*w/o, without; CI, confidence interval; ICU, Intensive Care Unit*.

Adverse outcomes affected both ICU and non-ICU-treated sepsis survivors, with higher rates in ICU-treated survivors compared to non-ICU-treated survivors (at least one adverse outcome: 55.9 vs. 46.2%, *p* < 0.001, Table 1). Adverse events also occurred more frequently in survivors of severe sepsis than in non-severe sepsis survivors of whom nearly half were affected (Table 1).

Among survivors without any pre-existing medical, psychological or physical impairment according to the study definition prior to the index hospitalization (*n* = 8,622), 29.8% had at least one adverse event in the first year post-sepsis, 19.4% of at risk-survivors became newly dependent on chronic care, 1.0% required new respiratory support, 1.7% started long-term dialysis, and 15.2% died within the first 12 months post-sepsis.

### Risk Factors for Adverse Outcomes

**Figure 2:**
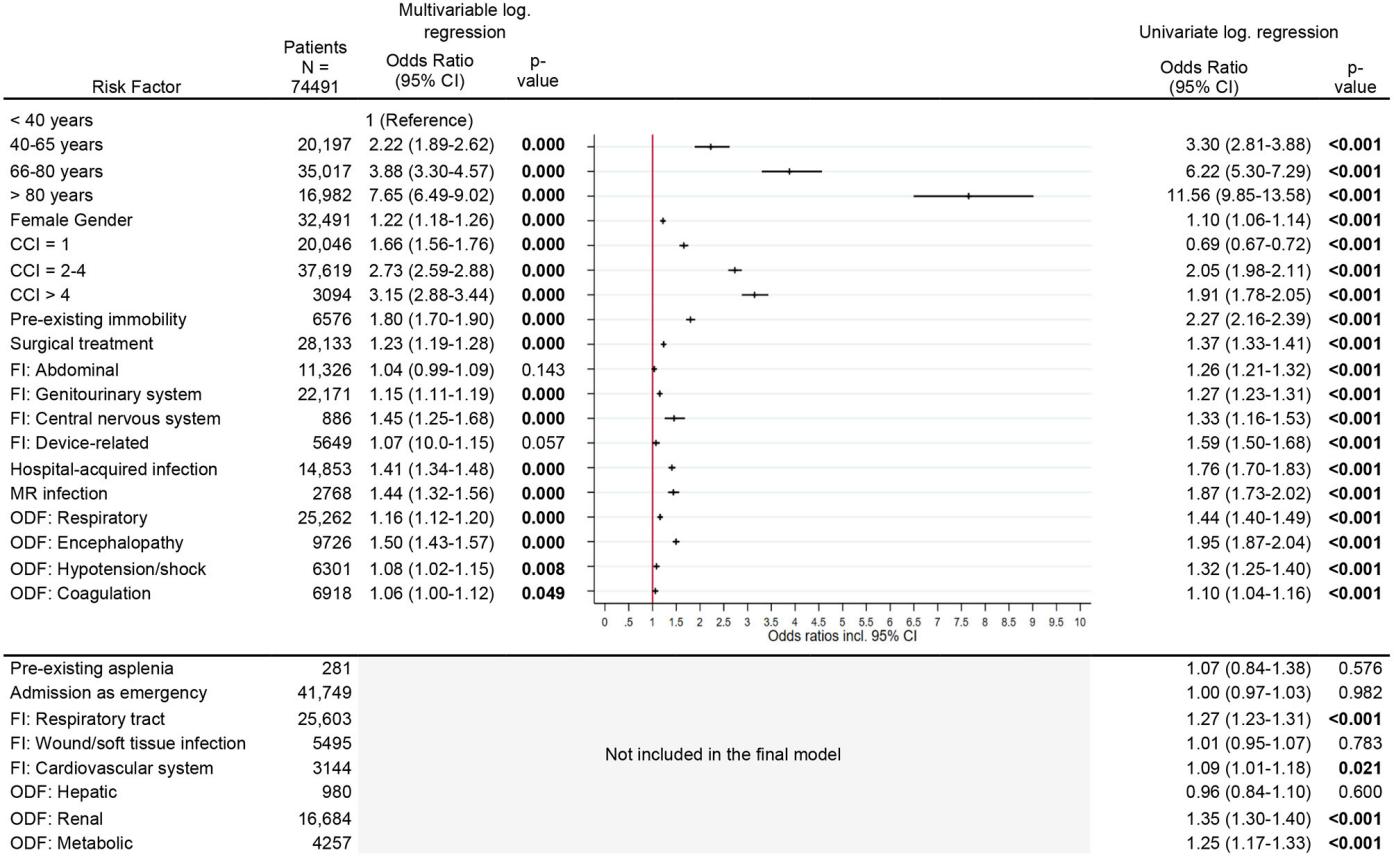
Risk factors for new chronic care dependency. Legend: CCI, Charlson Comorbidity Index; FI, Focus of infection; MR, Multi-resistant; ODF, Organ dysfunction.

**Figure 3:**
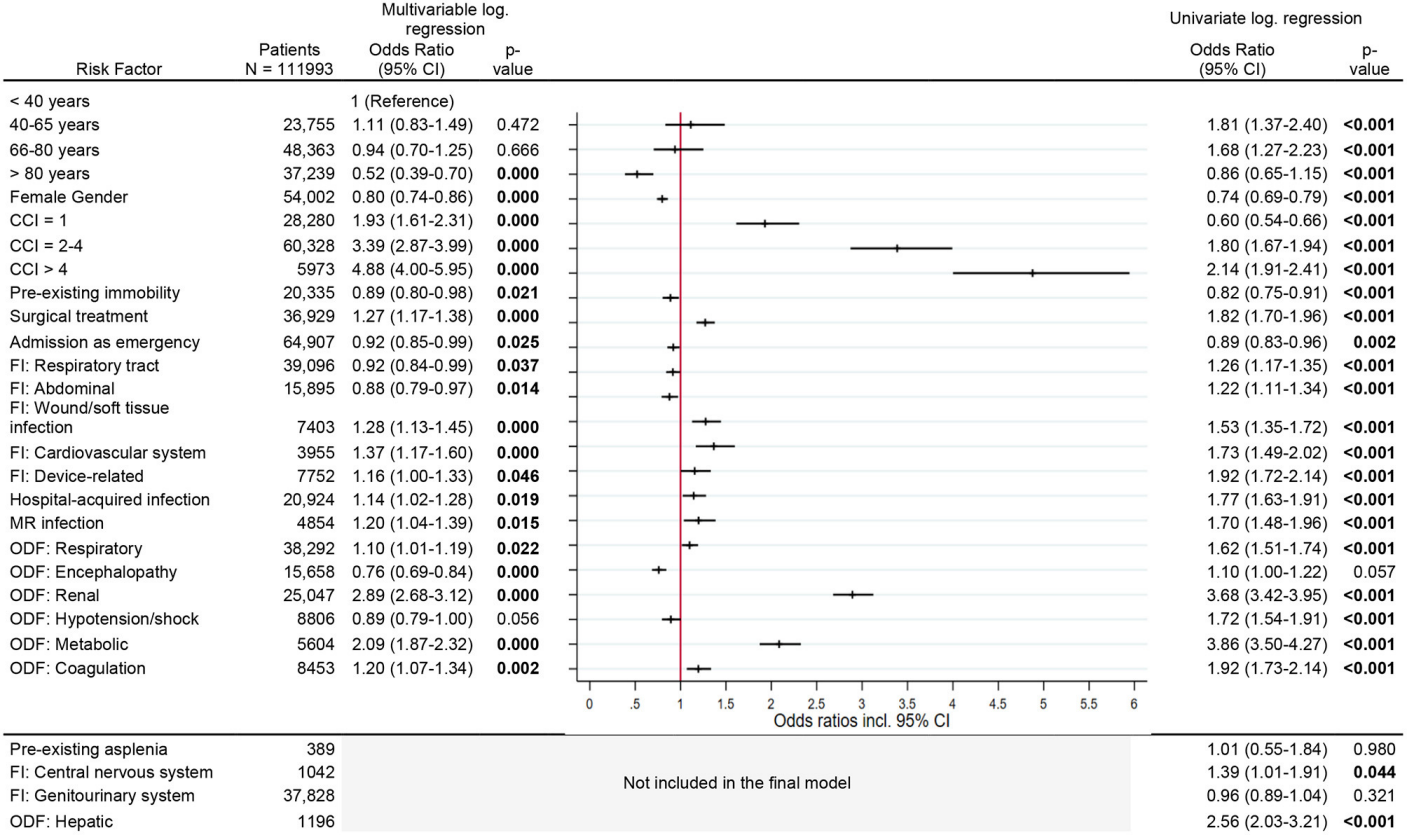
Risk factors for new diaysis dependency. Legend: CCI, Charlson Comorbidity Index; FI, Focus of infection; MR, Multi-resistant; ODF, Organ dysfunction.

**Figure 4:**
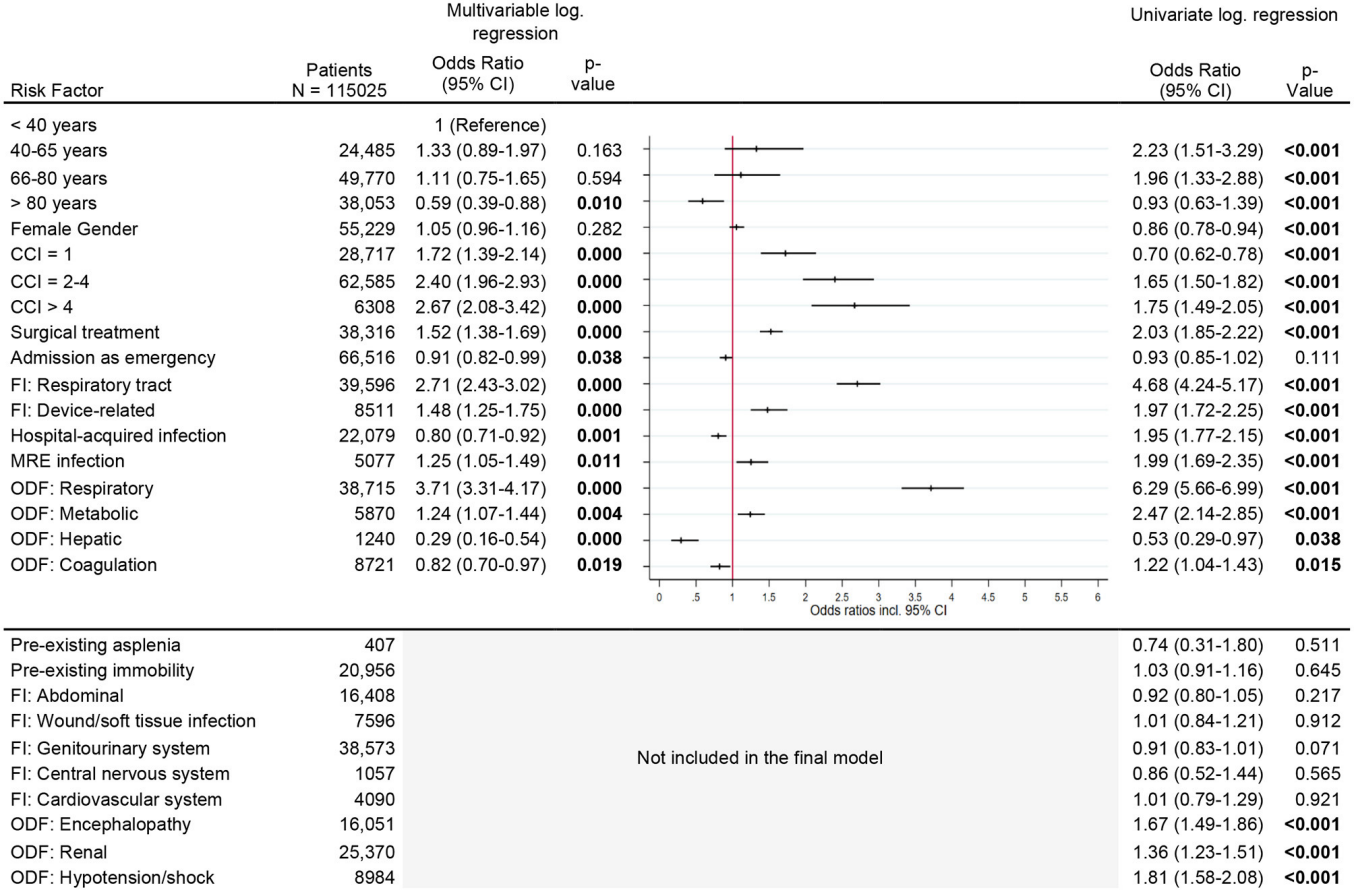
Risk factors for new respiratory support. Legend: CCI, Charlson Comorbidity Index; FI, Focus of infection; MR, Multi-resistant; ODF, Organ dysfunction.

**Figure 5:**
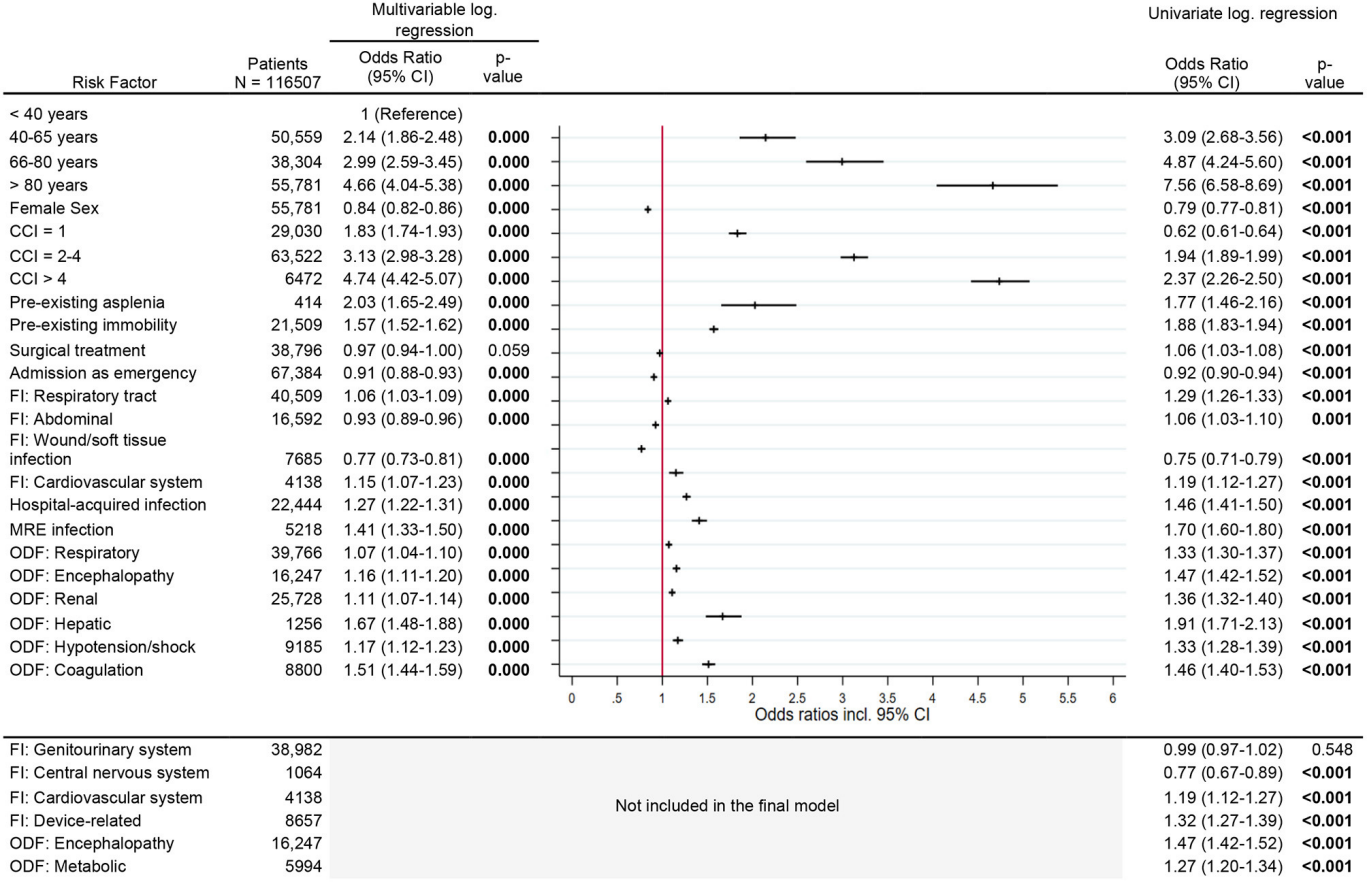
Risk factors for 12-months mortality. Legend: CCI, Charlson Comorbidity Index; FI, Focus of infection; MR, Multi-resistant; ODF, Organ dysfunction.

Results of univariate logistic regression analysis exploring bivariate associations are provided in Figures 2–**5**. In the following, we report the results of multivariable logistic regression analyses.

### Risk Factors for New Chronic Care Dependency

Considering all other risk factors, the risk for new chronic care dependency increased significantly with increasing age and comorbidity index. Patients older than 80 years and patients with a CCI > 4 had a several-fold increased OR [age >80 years: OR 7.7 (95% CI, 6.5–9.0)], CCI >4: OR 3.2 (95% CI, 2.9–3.4), Figure 2] for new chronic care dependency. Other risk factors were pre-existing immobility [OR 1.8 (95% CI, 1.7–1.9)], a central nervous system (CNS) infection or diagnosis of encephalopathy [OR 1.5 (95% CI, 1.3–1.7) and OR 1.5 (95% CI, 1.4–1.6), respectively] as well as a hospital-acquired infection or infection with multi-resistant organisms [OR 1.4 (95% CI, 1.3–1.5), OR 1.4 (95% CI, 1.3–1.6), respectively]. Female gender, surgical treatment, and other organ dysfunctions or infection sites had only slightly increased odds ratios (see Figure 2).

### Risk Factors for New Dialysis Dependency

The risk of becoming dialysis dependent increased with the comorbidity index [CCI > 4, OR 4.9 (95% CI, 4.0–6.0)]. Renal organ dysfunction during sepsis nearly trebled the risk for new dialysis dependency [OR 2.9 (95% CI, 2.7–3.1)], while metabolic organ dysfunction doubled the risk [OR 2.1 (95% CI, 1.9–2.3)]. Other risk factors were surgical treatment, soft tissue infection, cardiovascular or device related infection, infection with multi-resistant organisms and pre-existing immobility. The risk was significantly reduced in the age group >80 years [OR 0.5 (95% CI, 0.4–0.7)] compared to the reference age group <40 years and in female patients [OR 0.8 (95% CI, 0.7–0.9)] (Figure 3).

### Risk Factors for New Respiratory Support

The risk of needing long-term respiratory support increased with comorbidity index [CCI > 4, OR 2.7 (95% CI, 2.1–3.4)], respiratory infection as cause of sepsis [OR 2.7 (95% CI, 2.4–3.0)], and respiratory organ dysfunction [OR 3.7 (95% CI, 3.3–4.2)]. Additional factors, which increased the risk slightly, were surgical treatment, device-related infection, multi-resistant infection and metabolic organ dysfunction (Figure 4).

### Risk Factors for 12-Month Mortality

Risk factors for 12-month mortality were increasing age, with patients in the > 80 year age group having an OR of 4.7 (95% CI, 4.0–5.4), and increasing comorbidity index. Patients with a CCI > 4 had an OR of 4.7 (95% CI, 4.4–5.1). Other risk factors were asplenia [OR 2.0 (95% CI, 1.7–2.5)] and pre-existing immobility [OR 1.6 (95% CI, 1.5–1.6), *p* < 0.001] (Figure 5). Patients who had an emergency admission or sepsis due to a soft tissue infection had a reduced risk [OR 0.9 (95% CI, 0.9–0.9) and OR 0.8 (95% CI, 0.7–0.8), respectively]. Among different acute organ dysfunctions, hepatic dysfunction had the strongest association with long-term mortality [OR 1.7 (95% CI, 1.5–1.9), *p* < 0.001] (Figure 5).

## Discussion

In the first year after hospital discharge, 49% of 116,507 sepsis survivors developed severe adverse outcomes, including new dependencies on chronic care, dialysis or respiratory support, and death. The 12-month mortality rate (30.7%) was similar to data previously reported in the literature (24, 25) but considerably higher than in matched hospital controls without sepsis (26). One in three survivors experienced long-term functional disability requiring chronic care. The need for chronic care represents a surrogate for functional limitations which cannot be assessed in detail through health claims records. Nevertheless, our data indicate that functional limitations after sepsis are much more common than previously known (8).

New functional limitations cause severe disruption to peoples' daily lives, leading to dependency on formal and informal care (8), negative financial consequences (27) and reduced health-related quality of life (28), particularly in patients who also require chronic organ replacement therapies (29). At 12 months post-sepsis, 2.8% of survivors needed long-term dialysis and 1.6% were dependent on chronic respiratory support, which is at a similar scale as the incidence of prolonged mechanical ventilation in sepsis survivors with chronic critical illness (about 3.6%) (30). Although the proportions may be relatively low, the long-term impact of both, end-stage respiratory failure and chronic dialysis dependency need to be highlighted. Both conditions are associated with an increased risk of further health problems, including recurrent sepsis and cardiovascular morbidity, premature mortality and high health care costs. For instance, data from the UK showed that the annual costs for care following discharge from hospital after an episode of acute kidney injury were in the range of £179 million ($277 million USD) (31). These costs were mainly driven by the costs of long-term dialysis therapy. Finally, carers of patients with chronic diseases also report negative impacts on their physical health, mental well-being and financial/employment life (32).

Despite statistically significant differences, it is remarkable that the proportions of adverse outcomes are strikingly similar in survivors with non-severe sepsis and severe sepsis as well as in survivors of non-ICU-treated and ICU-treated sepsis. Even 29.8% of previously healthy survivors developed at least one adverse outcome and 19% needed new chronic care. These findings suggest that severe infections play a major role in the pathogenesis of adverse outcomes after sepsis (33).

In our study, pre-existing comorbidity was the most significant risk factor for all adverse outcomes after sepsis. For patients with more than four comorbidities, the odds of becoming dependent on chronic care or requiring long-term respiratory support increased more than 3-fold, while the odds of death within 1 year or need for long-term dialysis increased 5-fold. Higher age significantly increased the odds of new chronic care dependency and 12-month mortality, but not for new organ replacement therapies. The declining incidence rates of chronic critical illness in patients aged 80 years and older can potentially be explained by earlier mortality among otherwise eligible patients (30). However, it may also reflect age-dependent treatment preferences (34) and clinical decision-making, which is not reflected in health claims data.

Another important finding is that patients with central nervous infection and organ dysfunction had a greater risk of chronic care dependency compared to other infections and organ dysfunctions. Both may lead to chronic cognitive impairment (35) and loss of independence (8). Hepatic organ dysfunction was associated with the strongest increase in the odds of 12-month mortality among all acute organ dysfunctions. This is in line with several previous studies that observed an association between liver failure and a higher risk of short- and long-term mortality (19, 36).

Hospital-acquired infections increased the odds of chronic care dependency, chronic dialysis and 12-month mortality, but not of long-term respiratory support. Interestingly, hospital-acquired infections lowered the odds of long-term respiratory support (OR 0.8 [95% CI, 0.7 to 0.9]), contrary to the results from the univariate analyses [OR 2.0 (95% CI, 1.8–2.2)]. We hypothesize that early mortality of patients with hospital-acquired infections may be a possible explanation, but further research is needed to better understand the impact of hospital-acquired infection on need for long-term respiratory support.

A major strength of our study is the analysis of adverse long-term outcomes in a large population-based, unselected cohort of sepsis survivors. Using a health claims database, we were able to analyse complete healthcare records of inpatient and outpatient care for six consecutive years, including a 5 year period prior to the index hospitalization, in which we identified pre-existing asplenia. This allowed us to identify novel risk factors for adverse outcomes in sepsis survivors which have not been addressed in previous studies (12, 37, 38). Furthermore, we focused on three serious life changing adverse outcomes following sepsis, in addition to mortality.

This study also has several limitations. First, the identification of sepsis patients and the detection of risk factors and outcomes are dependent on the quality of coding in health claims databases. We expect that the diagnostic and procedural codes for long-term respiratory support and dialysis treatment are valid, given their high relevance for reimbursement of in- and outpatient services. Likewise, information on dependency on chronic care and vital status are routinely included in the health insurance patient data. The coding of risk factors such as comorbidities, however, cannot be fully validated. Since the introduction of the DRG system, comorbidity coding has improved. Not only the absolute number of coded secondary diagnoses, but also the mean number of Elixhauser diagnosis groups and records with more than five diagnosis groups have increased significantly (39). Studies from Denmark and the US found a high predictive value, but low sensitivity of comorbidity coding in hospital discharge data (40, 41), but it is unknown to which extent these observations apply to the German health system. Second, we could only capture pre-existing conditions which were routinely treated on an in- or outpatient basis. Therefore, not all potentially important risk factors could be identified (e.g., congenital asplenia). Third, we cannot make any conclusions about the underlying mechanisms leading to adverse outcomes after sepsis and are not able to determine causality between risk factors and long-term outcomes. Similarly, we cannot conclude causality between sepsis and long-term outcomes. Furthermore, we identified risk factors for the occurrence of adverse events, but cannot comment on the time course of adverse events or potential improvement due to the design of the study and the granularity of information captured in the health claims data. Fourth, we have no information about treatments administered, patient preferences or clinical decision making that may have impacted the provision of long-term organ replacement therapies. Fifth, we cannot determine the exact time of onset and clinical recovery from sepsis in individual patients, which may lead to varying follow-up periods. This particularly applies to patients who were discharged to other acute care facilities after their index hospitalization.

## Conclusion

Adverse outcomes affect a larger proportion of sepsis survivors than previously known. Both, patient- and infection-related risk factors contribute. Patients with pre-existing comorbidities have a considerably increased risk of developing adverse outcomes including death and should be regarded as particularly vulnerable. It is possible that they benefit from targeted interventions including vaccination, improved management of chronic diseases and other effective infection control measures. Given the significant associations between hospital-acquired and multi-resistant infections and adverse outcomes, prevention and control of hospital-acquired infections can be one key preventive measure to reduce the burden of long-term impairments beside high quality early sepsis care; management of pain, agitation, and delirium; and early mobilization to prevent or minimize muscle atrophy (6).

## Data Availability Statement

The data analyzed in this study is subject to the following licenses/restrictions: The authors confirm that the data utilized in this study cannot be made available in the article, the Supplementary Material, or in a public repository due to German data protection laws (“Bundesdatenschutzgesetz”, BDSG). Therefore, they are stored on a secure drive in the AOK Research Institute (WIdO), to facilitate replication of the results. Generally, access to data of statutory health insurance funds for research purposes is possible only under the conditions defined in German Social Law (SGB V § 287). Requests for data access can be sent as a formal proposal specifying the recipient and purpose of the data transfer to the appropriate data protection agency. Access to the data used in this study can only be provided to external parties under the conditions of the cooperation contract of this research project and after written approval by the sickness fund. For assistance in obtaining access to the data, please contact wido@wido.bv.aok.de. Requests to access these datasets should be directed to wido@wido.bv.aok.de.

## Author Contributions

MS, LW, and JS prepared and checked the data. PS supervised the statistical analyses. MS conducted the statistical analyses. MS, CF-S, and CH wrote the first draft of the manuscript. All authors revised the manuscript for important intellectual content, approved the final manuscript, and contributed to the idea and design of the study.

## Funding

German Innovations Fund of the Federal Joint Committee in Germany (G-BA) (Grant Number: 01VSF17010). The funder did not influence the design of the study, data collection, analysis, and interpretation, as well as the writing of the manuscript.

## Conflict of Interest

The authors declare that the research was conducted in the absence of any commercial or financial relationships that could be construed as a potential conflict of interest.

## Publisher's Note

All claims expressed in this article are solely those of the authors and do not necessarily represent those of their affiliated organizations, or those of the publisher, the editors and the reviewers. Any product that may be evaluated in this article, or claim that may be made by its manufacturer, is not guaranteed or endorsed by the publisher.
