# Supplementary material for: Occurrence and Risk Factors for New Dependency on Chronic Care, Respiratory Support, Dialysis and Mortality in the First Year After Sepsis
Source: Front Med (Lausanne). 2022 May 19;9:878337. doi: 10.3389/fmed.2022.878337 (PMC9162443; doi:10.3389/fmed.2022.878337)
Supplement: Supplementary file 1 [file Data_Sheet_1.docx]

Supplementary Material

**Occurrence and risk factors for new dependency on chronic care, mechanical ventilation, dialysis and mortality in the first year after sepsis**

Dr. Melissa Spoden^1^, Prof. Christiane S Hartog^2,3^, Prof. Peter Schlattmann^4^, PD Dr. Antje Freytag^5^, Lisa Wedekind^4^, Josephine Storch^5^, Prof. Konrad Reinhart^2^, Christian Günster^1^, Dr. Carolin Fleischmann-Struzek^6,7^.

1 Research Institute of the Local Health Care Funds (AOK), Rosenthaler Str. 31, 10178 Berlin, Germany

2 Department of Anesthesiology and Operative Intensive Care Medicine, Charité Universitätsmedizin Berlin, Charitépl. 1, 10117 Berlin, Germany

3 Klinik Bavaria, An d. Wolfsschlucht 1/2, 01731 Kreischa, Germany

4 Institute of Medical Statistics, Computer and Data Sciences, Jena University Hospital, Bachstraße 18, 07743 Jena, Germany

5 Institute of General Practice and Family Medicine, Jena University Hospital, Bachstraße 18, 07743 Jena, Germany

6 Center for Sepsis Control and Care, Jena University Hospital/Friedrich Schiller University Jena, Bachstraße 18, 07743 Jena, Germany

7 Institute of Infectious Diseases and Infection Control, Jena University Hospital, Am Klinikum 1, 07740 Jena, Germany

**Supplement Tab. 1: Baseline characteristics**

|  | **All survivors** | | **Survivors w/o pre-existing renal replacement therapy** | | **Survivors w/o pre-existing long term ventilation** | | **Survivors w/o pre-existing dependency on care** | |
| --- | --- | --- | --- | --- | --- | --- | --- | --- |
| **Index hospitalization** | **N** | **%** | **N** | **%** | **N** | **%** | **N** | **%** |
| N % | 116,507 | 73.0% | 1119,93 | 70.1% | 115,025 | 72.0% | 74,491 | 46.6% |
| Age, Mean (SD); Median (IQR) | 73.0 (13.3); 76 (16) | | 73.1 (13.3); 76 (16) | | 73.0 (13,3); 76 (16) | | 70.1 (13.3); 76 (17) | |
| Female sex, % | 55,781 | 47.9% | 54,002 | 48.2% | 55,229 | 48.0% | 32,491 | 43.6% |
| CCI, Mean (SD); Median (IQR) | 2.0 (1.4); 2 (2) | | 2.0 (1.4); 2 (2) | | 2.0 (1.4); 2 (2) | | 1.8 (1.4); 2 (2) | |
| Admission as emergency, % | 67,384 | 57.8% | 64,907 | 58.0% | 66,516 | 57.8% | 41,749 | 56.0% |
| Focus of infection, % |  |  |  |  |  |  |  |  |
| Respiratory tract | 40,509 | 34.8% | 39,096 | 34.9% | 39,596 | 34.4% | 25,603 | 34.4% |
| Abdominal | 16,592 | 14.2% | 15,895 | 14.2% | 16,408 | 14.3% | 11,326 | 15.2% |
| Wound/soft tissue infection | 7685 | 6.6% | 7403 | 6.6% | 7596 | 6.6% | 5495 | 7.4% |
| Genitourinary system | 38,982 | 33.5% | 37,828 | 33.8% | 38,573 | 33.5% | 22,171 | 29.8% |
| Central nervous system | 1064 | 0.9% | 1042 | 0.9% | 1057 | 0.9% | 886 | 1.2% |
| Vascular system | 4138 | 3.6% | 3955 | 3.5% | 4090 | 3.6% | 3144 | 4.2% |
| Device-related | 8657 | 7.4% | 7752 | 6.9% | 8511 | 7.4% | 5649 | 7.6% |
| Pregnancy associated infection | 103 | 0.1% | 103 | 0.1% | 103 | 0.1% | 102 | 0.1% |
| Hospital-acquired infection, % | 22,444 | 19.3% | 20,924 | 18.7% | 22,079 | 19.2% | 14,853 | 19.9% |
| Multi-resistant infection, % | 5218 | 4.5% | 4854 | 4.3% | 5077 | 4.4% | 2768 | 3.7% |
| Presence of any acute organ dysfunction, % | 64,517 | 55.4% | 62,130 | 55.5% | 63,309 | 55.0% | 41,010 | 55.1% |
| Number of organ dysfunctions, Mean (SD); Median (IQR) | 0.9 (1.1); 1 (1) | | 0.9 (1.1); 1 (1) | | 0.9 (1.1); 1 (1) | | 0.9 (1.1); 1 (1) | |
| Occurance of septic shock, % | 7888 | 6.8% | 7557 | 6.7% | 7710 | 6.7% | 5501 | 7.4% |
| ICU treatment, % | 32,238 | 27.7% | 30,742 | 27.4% | 31,570 | 27.4% | 23,068 | 31.0% |
| Mechanical ventilation, % | 19,311 | 16.6% | 18,645 | 16.6% | 18,787 | 16.3% | 14,166 | 19.0% |
| Renal replacement therapy, % | 7031 | 6.0% | 4846 | 4.3% | 6919 | 6.0% | 4954 | 6.7% |
| Surgical treatment, % | 38,796 | 33.3% | 36,929 | 33.0% | 38,316 | 33.3% | 28,133 | 37.8% |
| Palliative care, % | 1170 | 1.0% | 1155 | 1.0% | 1159 | 1.0% | 731 | 1.0% |
| Hospital length of stay, Mean (SD); Median (IQR) | 21.5 (21.0); 15 (17) | | 21.4 (20.9); 15 (17) | | 21.5 (21.0); 15 (17) | | 23.2 (22.9); 16 (20) | |
| Discharge disposition of survivors |  |  |  |  |  |  |  |  |
| - regular discharge | 84,482 | 72.5% | 81,164 | 72.5% | 83,519 | 72.6% | 54,847 | 73.6% |
| - other hospital | 14,265 | 12.2% | 13,594 | 12.1% | 13,973 | 12.1% | 10,319 | 13.9% |
| - rehabilitation | 6397 | 5.5% | 6193 | 5.5% | 6323 | 5.5% | 5230 | 7.0% |
| - nursing home | 9386 | 8.1% | 9153 | 8.2% | 9255 | 8.0% | 2699 | 3.6% |
| - hospice | 310 | 0.3% | 305 | 0.3% | 308 | 0.3% | 202 | 0.3% |
| - other | 1667 | 1.4% | 1584 | 1.4% | 1647 | 1.4% | 1194 | 1.6% |
|  |  |  |  |  |  |  |  |  |
| **Health status and health care use 12 months prior to index hospitalization** | | | | | | | | |
| Employed persons, % | 16,284 | 14.0% | 15,822 | 14.1% | 16,121 | 14.0% | 13,682 | 18.4% |
| Dependence on chronic care |  |  |  |  |  |  |  |  |
| - Nursing home residence, % | 12,595 | 10.8% | 12,215 | 10.9% | 12,450 | 10.8% | 0 | 0.0% |
| - Care level according to German care level system*, % | 41,629 | 35.7% | 39,522 | 35.3% | 40,790 | 35.5% | 0 | 0.0% |
| - Nursing home residence or care level, % | 42,016 | 36.1% | 39,899 | 35.6% | 41,161 | 35.8% | 0 | 0.0% |
| - Nursing care level according to German care level system, Mean (SD); Median (IQR) | 3.0 (1.0); 3 (2) | | 3.1 (1.0); 3 (2) | | 3.0 (1.0); 3 (2) | | - | |
| Pre-existing immobility, % | 21,509 | 18.5% | 20,335 | 18.2% | 20,956 | 18.2% | 6576 | 8.8% |
| Pre-existing long-term mechanical ventilation, % | 1482 | 1.3% | 1362 | 1.2% | 0 | 0.0% | 627 | 0.8% |
| Pre-existing renal replacement therapy, % | 4514 | 3.9% | 0 | 0.0% | 4394 | 3.8% | 2397 | 3.2% |
| Pre-existing asplenia, coded in the five years prior to sepsis index hospitalization, % | 414 | 0.4% | 389 | 0.3% | 407 | 0.4% | 281 | 0.4% |

**Supplement Tab. 2: Occurrence of adverse outcomes up to 6 months after sepsis**

| **Adverse Outcome** | All survivors | Patients w/o prior impairments | Severe sepsis | Non-severe sepsis | p-value | ICU-treated sepsis | Non-ICU-treated sepsis | p-value |
| --- | --- | --- | --- | --- | --- | --- | --- | --- |
| **No. of survivors** | 116,507 | 8,622 | 37,840 | 78,667 |  | 32,238 | 84,269 |  |
| Survivors with >=1 new adverse outcome among all survivors, % (95% CI) | 42.0 (41.7 - 42.2) | 26.2 (25.3 - 27.1) | 48.4 (47.9 - 48.9) | 38.8 (38.5 - 39.2) | <0.001 | 50.0 (49.5 - 50.5) | 38.9 (38.6 - 39.2) | <0.001 |
| New onset chronic care dependency among survivors w/o prior chronic care dependency, % (95% CI) | 28.5 (28.2 - 28.9) | 17.5 (16.7 - 18.3) | 32.2 (31.7 - 32.8) | 26.7 (26.3 - 27.1) | <0.001 | 34.6 (34.0 - 35.2) | 25.8 (25.4 - 26.2) | <0.001 |
| New onset dialysis dependency among survivors w/o prior dialysis, % (95% CI) | 2.2 (2.1 - 2.3) | 1.4 (1.2 - 1.7) | 3.5 (3.3 - 3.7) | 1.6 (1.5 - 1.7) | <0.001 | 3.9 (3.7 - 4.1) | 1.6 (1.5 - 1.6) | <0.001 |
| New onset respiratory support among survivors w/o prior respiratory support, % (95% CI) | 1.2 (1.2 - 1.3) | 0.8 (0.6 – 1.0) | 1.9 (1.8 - 2.0) | 0.9 (0.8 - 1.0) | <0.001 | 2.5 (2.4 - 2.7) | 0.7 (0.7 - 0.8) | <0.001 |
| 12-months mortality among hospital survivors, % (95% CI) | 23.2 (23 - 23.5) | 11.3 (10.6 - 11.9) | 26.5 (26.1 - 27.0) | 21.7 (21.4 - 21.9) | <0.001 | 24.7 (24.3 - 25.2) | 22.7 (22.4 - 22.9) | <0.001 |

**Supplement: Definitions**

**A - Sepsis definitions**

ICD-10-German Modification (GM) Codes: A02.1 - Salmonella sepsis, A20.0 - Bubonic plague, A20.7 - Septicaemic plague, A21.7 - Generalized tularaemia, A22.7 - Anthrax sepsis, A24.1 - Acute or fulminating melioidosis, A26.7 - Erysipelothrix sepsis, A28.2 - Extraintestinal yersiniosis, A32.7 - Listerial sepsis, A39.1 - Waterhouse-Friderichsen syndrome, A39.2-acute meningococcal sepsis, A39.3-chronic meningogoccal sepsis, A39.4 - Meningococcaemia, unspecified, A40.-Streptococcal sepsis, A41. - Other sepsis, A42.7 - Actinomycotic sepsis, A48.3 - Toxic shock syndrome, A49.9-Bacterial infection, unspecified, A54.8-Other gonococcal infections, B00.7 - Disseminated herpesviral disease, B37.6 - Candidal endocarditis, B37.7 - Candidal sepsis, B49 - Unspecified mycosis, O75.3 - Other infection during labour, O85 - other puerperal infections, R65.0 - Systemic Inflammatory Response Syndrome of infectious origin without organ failure, R65.1 - Systemic Inflammatory Response Syndrome of infectious origin with organ failure, R57.2 - Septic shock

**B - Subgroups definitions**

Severe sepsis at index hospitalization

ICD-10-GM Codes: R65.1 - Systemic Inflammatory Response Syndrome of infectious origin with organ failure, ICD-10-GM Codes: R57.2 - Septic shock

ICU-treated sepsis at index hospitalization

Operation and Procedural Codes (OPS) Codes: 8-980 - Intensive care complex treatment, 8-98f - Costly intensive care complex treatment (basic procedure), 8-98d - Intensive care complex treatment in childhood (basic procedure), 8-98c - Intensive care complex treatment in childhood

Patients without prior impairments in the 12 months prior to index hospitalization

No cognitive, medical or psychological impairment:

Cognitive impairment

ICD-10-GM Codes: F06.7 - Mild cognitive disorder, U51.- - Impairment of cognitive function, R41.0 - Disorientation, unspecified, F00* - Dementia in Alzheimer disease, F01 - Vascular dementia, F02* - Dementia in other diseases classified elsewhere, F03 - Unspecified dementia, F04 - Organic amnesic syndrome, not induced by alcohol and other psychoactive substances, F05 - Delirium, not induced by alcohol and other psychoactive substances, F06.9 - Unspecified organic mental disorder due to brain damage and dysfunction and to physical disease, F07.8 - Other organic personality and behavioural disorders due to brain disease, damage and dysfunction, F07.9 - Unspecified organic personality and behavioural disorder due to brain disease, damage and dysfunction, G30 - Alzheimer disease, G31.0 - Circumscribed brain atrophy, G31.1 - Senile degeneration of brain, not elsewhere classified, G31.9 - Degenerative disease of nervous system, unspecified, G32* - Other degenerative disorders of nervous system in diseases classified elsewhere

Psychological impairment

PTSD

ICD-10-GM Codes: F43 - Reaction to severe stress, and adjustment disorders, F43.0 - Acute stress reaction, F43.1 - Post-traumatic stress disorder, F43.2 - Adjustment disorders, F43.8 - Other reactions to severe stress, F43.9 - Reaction to severe stress, unspecified

Depression

ICD-10-GM Codes: F32 - Depressive episode, F33 - Recurrent depressive disorder, F34.1 - Dysthymia, F38 - Other mood [affective] disorders, F41.2 - Mixed anxiety and depressive disorder, F06.3 - Organic mood [affective] disorders

Anxiety

ICD-10-GM Codes: F40 - Phobic anxiety disorders, F41 - Other anxiety disorders, F06.4 - Organic anxiety disorder

Sleeping disorders

ICD-10-GM Codes: F51 - Nonorganic sleep disorders, G47 - Sleep disorders

Substance abuse

ICD-10-GM Codes: F10 - Mental and behavioural disorders due to use of alcohol, F11 - Mental and behavioural disorders due to use of opioids, F12 - Mental and behavioural disorders due to use of cannabinoids, F13 - Mental and behavioural disorders due to use of sedatives or hypnotics, F14 - Mental and behavioural disorders due to use of cocaine, F15 - Mental and behavioural disorders due to use of other stimulants, including caffeine, F16 - Mental and behavioural disorders due to use of hallucinogens, F17 - Mental and behavioural disorders due to use of tobacco, F18 - Mental and behavioural disorders due to use of volatile solvents, F19 - Mental and behavioural disorders due to multiple drug use and use of other psychoactive substances

Medical impairment

Respiratory dysfunction

ICD-10-GM Codes: J96 - Respiratory failure, not elsewhere classified, J98 - Other respiratory disorders, R06.0 - Dyspnoea, J80 - Adult respiratory distress syndrome

Cardiovascular diseases

Coronary heart disease and myocardial infarction

ICD-10-GM Codes: I20 - Angina pectoris, I21 - Acute myocardial infarction, I22 - Subsequent myocardial infarction, I24 - Other acute ischaemic heart diseases, I25 - Chronic ischaemic heart disease

Cardiomyopathy

ICD-10-GM Codes: I42 – Cardiomyopathy

Heart failure

ICD-10-GM Codes: I50 - Heart failure

Cardiac arrhythmias

ICD-10-GM Codes: I47 - Paroxysmal tachycardia, I48 - Atrial fibrillation and flutter, I49 - Other cardiac arrhythmias

Cerebrovascular diseases

ICD-10-GM Codes: I63 - Cerebral infarction, I64 - Stroke, not specified as haemorrhage or infarction, I65 - Occlusion and stenosis of precerebral arteries, not resulting in cerebral infarction, I66 - Occlusion and stenosis of cerebral arteries, not resulting in cerebral infarction

Renal diseases

ICD-10-GM Codes: N17 - Acute renal failure, N18 - Chronic kidney disease, N19 - Unspecified kidney failure

Hepatic diseases

ICD-10-GM Codes: K72.1 - Chronic hepatic failure

Metabolic diseases

Diabetes mellitus

ICD-10-GM Codes: E11 - Type 2 diabetes mellitus, E12 - Malnutrition-related diabetes mellitus, E13 - Other specified diabetes mellitus, E14 - Unspecified diabetes mellitus

Other metabolic diseases

ICD-10-GM Codes: E27 - Other disorders of adrenal gland, E35* - Disorders of endocrine glands in diseases classified elsewhere, E34.9 - Endocrine disorder, unspecified, E23 - Hypofunction and other disorders of pituitary gland

Anaemia

ICD-10-GM Codes: D50 - Iron deficiency anaemia, D51 - Vitamin B12 deficiency anaemia, D52 - Folate deficiency anaemia, D53 - Other nutritional anaemias, D63 - Anaemia in chronic diseases classified elsewhere, D64.9 - Anaemia, unspecified

Neuromuscular/musculoskeletal diseases

ICUAW/CIP/CIM

ICD-10-GM Codes: G62.8 - Critical illness polyneuropathy, G72.8 - Critical illness myopathy

Dysphagia

ICD-10-GM Codes: R13 - Dysphagia

Voice disorders

ICD-10-GM Codes: R49 - Voice disturbances

Contractures

ICD-10-GM Codes: M62.4 - Contracture of muscle, M24.5 - Contracture of joint, M25.6 - Stiffness of joint, not elsewhere classified, M21.62 - Acquired Pes equinus

Immobility

ICD-10-GM Codes: R26.2 - Difficulty in walking, not elsewhere classified, R26.3 - Immobility, R29.6 - Tendency to fall, not elsewhere classified, Z99.3 - Dependence on wheelchair, Z74.0 - Need for assistance due to reduced mobility

Decubitus

ICD-10-GM Codes: L89 - Decubitus ulcer and pressure area

Complications of tracheostomy

Complications of the tracheostoma

ICD-10-GM Codes: Z43.0 - Attention to tracheostomy, Z93.0 - Tracheostomy status, J95.0 - Tracheostomy malfunction

Tracheal stenoses

ICD-10-GM Codes: J95.5 - Postprocedural subglottic stenosis J95.81 - Tracheal stenosis following a procedure, J38.6 - Stenosis of larynx, J39.8 - Acquired tracheal stenosis

Urogenital diseases

Incontinence

ICD-10-GM Codes: R32 - Unspecified urinary incontinence, N39.3 - Stress incontinence, N39.4 - Other specified urinary incontinence, R15 - Faecal incontinence

Sexual disorders

ICD-10-GM Codes: F52 - Sexual dysfunction, not caused by organic disorder or disease

Urethral stricture

ICD-10-GM Codes: N99.1 - Postprocedural urethral stricture

Sensory disorders

Vestibular disorders

ICD-10-GM Codes: R42 - Dizziness and giddiness

Hearing disorder

ICD-10-GM Codes: H90 - Conductive and sensorineural hearing loss, H91 - Other hearing loss, H93 - Other disorders of ear, not elsewhere classified

Taste and smelling disorders

ICD-10-GM Codes: R43 - Disturbances of smell and taste

Impairment of nutrition

ICD-10-GM Codes: E41 - Nutritional marasmus, E43 - Unspecified severe protein-energy malnutrition, E44 - Protein-energy malnutrition of moderate and mild degree, E46 - Unspecified protein-energy malnutrition, R63.0 - Anorexia, R63.3 - Feeding difficulties and mismanagement, R63.4 - Abnormal weight loss, R63.6 - Insufficient intake of food and water, R63.8 - Other symptoms and signs concerning food and fluid intake, R64 - Cachexia

Multidrug-resistant infections

ICD-10-GM Codes: U80.-! - Gram-positive bacteria with specified antibiotic resistance, requiring special therapeutic or hygienic measures, U81.-! - Gram-negative bacteria with specified antibiotic resistance, requiring special therapeutic or hygienic measures, U82 - Mycobacteria with resistance against TB drugs (first line), U83 - Candida with resistance against Fluconazole and Voriconazole, U84 - Herpes virus with restistance against antivirals

Chronic pain

ICD-10-GM Codes: R52.1 - Chronic intractable pain, R52.2 - Other chronic pain, R52.9 - Pain, unspecified, F45.4 - Persistent somatoform pain disorder, F45.41 - Chronic pain disorder associated with psychological and behavioural factors, G54.6 - Phantom limb syndrome with pain

Fatigue

ICD-10-GM Codes: R53 - Malaise and fatigue, G93.3 - Chronic fatigue syndrome

**C - Definition of outcomes**

Long-term mechanical ventilation

ICD-10-GM Codes: Z99.0 - Dependence on aspirator, Z99.1 - Dependence on respirator

OPS Codes: 8-713 - Mechanical ventilation and respiratory support in adults, 8-716 - Setting up of home mechanical ventilation, 8-718 - Weaning from mechanical ventilation

Renal replacement therapy

ICD-10-GM Codes: Z99.2 - Dependence on renal dialysis, Z49 - Care involving dialysis

OPS Codes: 8-853 - Haemofiltration, 8-854 - Haemodialysis, 8-855 - Haemodiafiltration, 8-857 - Peritoneal dialysis, 8-85a - Dialysis procedure due to a functional failure and failure of a kidney transplant

**D - Potential risk factors**

Age in years at index hospitalization

<40 years

40-65 years

66-80 years

>80 years

Sex

Male, female

Comorbidities in the 12 months prior to index hospitalization

defined according to Charlson Comorbidity Index[20]

Pre-existing asplenia, coded in the five years prior to sepsis index hospitalization

ICD-10-GM Codes: Q89.0 - Asplenia (congenital), Q89.01 - Asplenia (congenital)

OPS Codes: 5-4131 - Splenectomy, total

Pre-existing immobility, coded in the 12 months prior to sepsis index hospitalization

ICD-10-GM Codes: R26.2 - Difficulty in walking, not elsewhere classified, R26.3 - Immobility, R29.6 - Tendency to fall, not elsewhere classified, Z99.3 - Dependence on wheelchair, Z74.0 - Need for assistance due to reduced mobility

Focus of infection at index hospitalization

Respiratory tract

ICD-10-GM Codes: J01 - Acute sinusitis, J02 - Acute pharyngitis, J03 - Acute tonsillitis, J04 - Acute laryngitis and tracheitis, J06 - Acute upper respiratory infections of multiple and unspecified sites, J05 - Acute obstructive laryngitis [croup] and epiglottitis, J09 - Influenza due to identified zoonotic or pandemic influenza virus, J10 - Influenza due to identified seasonal influenza virus, J11 - Influenza, virus not identified, J12 - Viral pneumonia, not elsewhere classified, J13 - Pneumonia due to Streptococcus pneumoniae, J14 - Pneumonia due to Haemophilus influenzae, J15 - Bacterial pneumonia, not elsewhere classified, J16 - Pneumonia due to other infectious organisms, not elsewhere classified, J17 - Pneumonia in diseases classified elsewhere, J18 - Pneumonia, organism unspecified, J20 - Acute bronchitis, J21 - Acute bronchiolitis, J22 - Unspecified acute lower respiratory infection, J44.0 - Chronic obstructive pulmonary disease with acute lower respiratory infection, J44.1 - Chronic obstructive pulmonary disease with acute exacerbation, unspecified, J86 - Pyothorax, J85 - Abscess of lung and mediastinum, A15 - Respiratory tuberculosis, bacteriologicallyorhistologically confirmed, A16 - Respiratory tuberculosis, not confirmed bacteriologically or histologically, U69.00 - Hospital-acquired pneumonia in other diseases classified elsewhere, A36 - Diphtheria, A37 - Whooping cough, B38 - Coccidioidomycosis, B39 - Histoplasmosis

Abdominal infections

ICD-10-GM Codes: A00 - Cholera, A01 - Typhoid and paratyphoid fevers, A02 - Other salmonella infections, A03 - Shigellosis, A04 - Other bacterial intestinal infections, A05 - Other bacterial foodborne intoxications, not elsewhere classified, A06 - Amoebiasis, A07 - Other protozoal intestinal diseases, A08 - Viral and other specified intestinal infections, A09 - Other gastroenteritis and colitis of infectious and unspecified origin, K35 - Acute appendicitis, K37 - Unspecified appendicitis, K36 - Other appendicitis, K5702 - Diverticular disease of small intestine with perforation and abscess without bleeding, K5703 - Diverticular disease of small intestine with perforation and abscess with bleeding, K5712 - Diverticular disease of small intestine without perforation or abscess without bleeding, K57.13 - Diverticular disease of small intestine without perforation or abscess wit bleeding, K57.22 - Diverticular disease of large intestine with perforation and abscess without bleeding, K57.23 - Diverticular disease of large intestine with perforation, abscess and bleeding, K57.32 - Diverticular disease of large intestine without perforation or abscess wihout bleeding, K5733 - Diverticular disease of large intestine without perforation or abscess wit bleeding, K5742 - Diverticular disease of both small and large intestine with perforation and abscess without bleeding, K5743 - Diverticular disease of both small and large intestine with perforation, abscess and bleeding, K5752 - Diverticular disease of both small and large intestine without perforation or abscess or bleeding, K5753 - Diverticular disease of both small and large intestine without perforation or abscess with bleeding, K5782 - Diverticular disease of intestine, part unspecified, with perforation and abscess without bleeding, K5783 - Diverticular disease of intestine, part unspecified, with perforation, abscess and bleeding, K5792 - Diverticular disease of intestine, part unspecified, without perforation, abscess or bleeding, K5793 - Diverticular disease of intestine, part unspecified, without perforation or abscess with bleeding, K61 - Abscess of anal and rectal regions, K65 - Peritonitis, K67 - Disorders of peritoneum in infectious diseases classified elsewhere, K63.0 - Abscess of intestine, K63.1 - Perforation of intestine (nontraumatic), K75.0 - Abscess of liver, K75.1 - Phlebitis of portal vein, K81.0 - Cholecystitis, K77.0 - Liver disorders in infectious and parasitic diseases classified elsewhere, U69.40! - Recurrent infection due to Clostridium difficile

Wound/soft tissue infection

ICD-10-GM Codes: A46 - Erysipelas, B47 - Mycetoma, L03 - Phlegmon, L04 - Acute lymphadenitis, L08 - Other local infections of skin and subcutaneous tissue, L05 - Pilonidal cyst, B00 - Herpesviral [herpes simplex] infections, B07 - Viral warts, B08 - Other viral infections characterized by skin and mucous membrane lesions, not elsewhere classified, B09 - Unspecified viral infection characterized by skin and mucous membrane lesions, H05.0 - Acute inflammation of orbit, H60.2 - Malignant otitis externa, H70.0 - Acute mastoiditis, J36 - Peritonsillar abscess, J39.0 - Retropharyngeal and parapharyngeal abscess, J39.1 - Other abscess of pharynx, L02 - Cutaneous abscess, furuncle and carbuncle

Genitourinary system infection

ICD-10-GM Codes: N10 - Acute tubulo-interstitial nephritis, N15.1 - Renal and perinephric abscess, N15.9 - Renal tubulo-interstitial disease, unspecified, N34 - Urethritis and urethral syndrome, N30 - Cystitis, N39.0 - Urinary tract infection, site not specified, N41 - Inflammatory diseases of prostate, N45 - Orchitis and epididymitis, N48.2 - Other inflammatory disorders of penis, N49 - Inflammatory disorders of male genital organs, not elsewhere classified, N70 - Salpingitis and oophoritis, N71 - Inflammatory disease of uterus, except cervix, N72 - Inflammatory disease of cervix uteri, N73 - Other female pelvic inflammatory diseases, N74 - Female pelvic inflammatory disorders in diseases classified elsewhere, N75 - Diseases of Bartholin gland, N76 - Other inflammation of vagina and vulva, N77 - Vulvovaginal ulceration and inflammation in diseases classified elsewhere, N61 - Inflammatory disorders of breast, N98.0 - Infection associated with artificial insemination, A59 - Trichomoniasis, A55 - Chlamydial lymphogranuloma (venereum), A56 - Other sexually transmitted chlamydial diseases

Central nervous system infection

ICD-10-GM Codes: A39 - Meningococcal infection, G00 - Bacterial meningitis, not elsewhere classified, G01 - Meningitis in bacterial diseases classified elsewhere, G02 - Meningitis in other infectious and parasitic diseases classified elsewhere, G03 - Meningitis due to other and unspecified causes, G04 - Encephalitis, myelitis and encephalomyelitis, G05* - Encephalitis, myelitis and encephalomyelitis in diseases classified elsewhere, G06 - Intracranial and intraspinal abscess and granuloma, G07* - Intracranial and intraspinal abscess and granuloma in diseases classified elsewhere, G08 - Intracranial and intraspinal phlebitis and thrombophlebitis, A17+ - Tuberculosis of nervous system, A81 - Atypical virus infections of central nervous system, A83 - Mosquito-borne viral encephalitis, A84 - Tick-borne viral encephalitis, A85 - Other viral encephalitis, not elsewhere classified, A86 - Unspecified viral encephalitis, A87 - Viral meningitis, A88 - Other viral infections of central nervous system, not elsewhere classified, A89 - Unspecified viral infection of central nervous system

Cardiovascular system infection

ICD-10-GM Codes: I32 - Pericarditis in diseases classified elsewhere, I33 - Acute and subacute endocarditis, I39 - Endocarditis and heart valve disorders in diseases classified elsewhere, I40 - Acute myocarditis, I41 - Myocarditis in diseases classified elsewhere, I80 - Thombosis, phlebitis and thrombophlebitis, I38 - Endocarditis, valve unspecified, I98.1 - Cardiovascular disorders in other infectious and parasitic diseases classified elsewhere

Device-related infections

ICD-10-GM Codes: T82.6 - Infection and inflammatory reaction due to cardiac valve prosthesis, T82.7 - Infection and inflammatory reaction due to other cardiac and vascular devices , implants and grafts, T83.5 - Infection and inflammatory reaction due to prosthetic device, implant and graft in urinary system, T83.6 - Infection and inflammatory reaction due to prosthetic device, implant and graft in genital tract, T84.5 - Infection and inflammatory reaction due to internal joint prosthesis, T84.6 - Infection and inflammatory reaction due to internal fixation device [any site], T84.7 - Infection and inflammatory reaction due to other internal orthopaedic prosthetic devices, implants and grafts, T85.7 - Infection and inflammatory reaction due to other internal prosthetic devices, implants and grafts

Pregnancy associated infection

ICD-10-GM Codes: O75.3 - Other infection during labour, O85 - Puerperal fever, O030 - Spontaneous abortion; Incomplete, complicated by genital tract and pelvic infection, O035 - Spontaneous abortion; Complete or unspecified, complicated by genital tract and pelvic infection, O040 - Medical abortion; Incomplete, complicated by genital tract and pelvic infection, O045 - Medical abortion; Complete or unspecified, complicated by genital tract and pelvic infection, O050 - Other abortion; Incomplete, complicated by genital tract and pelvic infection, O055 - Other abortion; Complete or unspecified, complicated by genital tract and pelvic infection, O060 - unspecified abortion; Incomplete, complicated by genital tract and pelvic infection, O065 - Unspecified abortion; Complete or unspecified, complicated by genital tract and pelvic infection, O070--O07.5 - Failed medical abortion, complicated by genital tract and pelvic infection, O075 - Other and unspecified failed attempted abortion, complicated by genital tract and pelvic infection, O08.0 - Genital tract and pelvic infection following abortion and ectopic and molar pregnancy, O86 - Other puerperal infections, O23 - Infections of genitourinary tract in pregnancy, O41.1 - Infection of amniotic sac and membranes, O88.3 - Obstetric pyaemic and septic embolism, O91 - Infections of breast associated with childbirth, O98 - Maternal infectious and parasitic diseases classifiable elsewhere but complicating pregnancy, childbirth and the puerperium,

Hospital-acquired infections at index hospitalization

ICD-10-GM Codes: T82.6 - Infection and inflammatory reaction due to cardiac valve prosthesis, T82.7 - Infection and inflammatory reaction due to other cardiac and vascular devices, implants and grafts, T84.5 - Infection and inflammatory reaction due to internal joint prosthesis, T84.6 - Infection and inflammatory reaction due to internal fixation device [any site], T84.7 - Infection and inflammatory reaction due to other internal orthopaedic prosthetic devices, implants and grafts, T85.72 - Infection and inflammatory reaction due to internal prosthetic devices, implants and grafts in the central nervous system, T85.73 - Infection and inflammatory reaction due to prosthetic devices or implants of the mamma, T85.75 - Infection and inflammatory reaction due to internal prosthetic devices, implants or grafts of the hepatobiliary system or pancreas, T85.76 - Infection and inflammatory reaction due to internal prosthetic devices, implants or grafts of the other gastrointestinal system, T85.78 - Infection and inflammatory reaction due to other internal prosthetic devices, implants and grafts, O86.0 - Infection of obstetric surgical wound, T83.5 - Infection and inflammatory reaction due to prosthetic device, implant and graft in urinary system, T83.6 - Infection and inflammatory reaction due to prosthetic device, implant and graft in genital tract, A04.7 - Enterocolitis due to Clostridium difficile, U69.40! - Recurrent infection due to Clostridium difficile, T80.2 - Infections following infusion, transfusion and therapeutic injection, T82.7 - Infection and inflammatory reaction due to other cardiac and vascular devices, implants and grafts, T81.4 - Infection following a procedure, not elsewhere classified, T85.71 - Infection and inflammatory reaction due to peritoneal dialysis catheter, T85.74 - Infection and inflammatory reaction due to percutaneous endoscopic gastrostomy/jejunostomy,T88.0 - Infection following immunization, U69.00 - Hospital-acquired pneumonia in patients aged 18 years or older

Multi-resistant infections at index hospitalization

ICD-10-GM Codes: U80.! - Grampositive bacteria with specified antibiotic resistance, requiring special therapeutic or hygienic measures, U81.! - Gram - negative bacteria with specified antibiotic resistance, requiring special therapeutic or hygienic measures, U82.! - Mycobacteria with resistance against TB drugs (first line), U83.! - Candida with resistance against Fluconazole and Voriconazole, U84.! - Herpes virus with restistance against antivirals, U85! - Human Immunodeficiency Virus with resistance against antivirals or proteinase – inhibitors

OPS Codes: 8-987 - Complex treatment in the case of colonisation or infection with multidrug-resistant pathogens [MDR]

Organ dysfunction at index hospitalization

Cardiovascular dysfunction/shock

ICD-10-GM Codes: I95.9 - Hypotension, unspecified, R57.8 - Other shock, R57.9 - Shock, unspecified, R57.2 - Septic shock

Respiratory dysfunction

ICD-10-GM Codes: J96. - Respiratory failure, not elsewhere classified, J80 - Adult respiratory distress syndrome, J98.4 - Other disorders of lung, R06.0 - Dyspnoea, R06.8 - Other and unspecified abnormalities of breathing

Encephalopathy

ICD-10-GM Codes: F05 - Delirium, not induced by alcohol and other psychoactive substances, G93.1 - Anoxic brain damage, not elsewhere classified, G93.4 - Encephalopathy, unspecified, R40 - Somnolence, stupor and coma

Renal dysfunction

ICD-10-GM Codes: N17. - Acute renal failure, N19 - Unspecified kidney failure,

Metabolic dysfunction

ICD-10-GM Codes: E87.2 - Acidosis

Abnormal coagulation

ICD-10-GM Codes: Coagulation D65 - Disseminated intravascular coagulation [defibrination syndrome], D68.8 - Other specified coagulation defects, D68.9 - Coagulation defect, unspecified, D69.5 - Secondary thrombocytopenia, D69.6 - Thrombocytopenia, unspecified,

Hepatic dysfunction

ICD-10-GM Codes: K72.0 Acute and subacute hepatic failure, K72.7 - Hepatic encephalopathy and hepatic coma, K72.9 - Hepatic failure, unspecified, K76.2 - Central haemorrhagic necrosis of liver, K76.3 - Infarction of liver,

Other organ dysfunction

ICD-10-GM Codes: R65.1 - Systemic Inflammatory Response Syndrome of infectious origin with organ complications

Emergency admission

Admission category 4

Surgical treatment

OPS Codes: Any OPS Code from Chapter 5 (surgical procedures)
